# Disentangling collective coupling in vibrational polaritons with double quantum coherence spectroscopy

**DOI:** 10.1063/5.0239877

**Published:** 2024-12-28

**Authors:** Thomas Schnappinger, Cyril Falvo, Markus Kowalewski

**Affiliations:** 1 Department of Physics, Stockholm University, AlbaNova University Center, SE-10691 Stockholm, Sweden; 2 Université Paris-Saclay, CNRS, Institut des Sciences Moléculaires d’Orsay, 91405 Orsay, France; 3 Université Grenoble-Alpes, CNRS, LIPhy, 38000 Grenoble, France

## Abstract

Vibrational polaritons are formed by strong coupling of molecular vibrations and photon modes in an optical cavity. Experiments have demonstrated that vibrational strong coupling can change molecular properties and even affect chemical reactivity. However, the interactions in a molecular ensemble are complex, and the exact mechanisms that lead to modifications are not fully understood yet. We simulate two-dimensional infrared spectra of molecular vibrational polaritons based on the double quantum coherence technique to gain further insight into the complex many-body structure of these hybrid light–matter states. Double quantum coherence uniquely resolves the excitation of hybrid light–matter polaritons and allows one to directly probe the anharmonicities of the resulting states. By combining the cavity Born–Oppenheimer Hartree–Fock ansatz with a full quantum dynamics simulation of the corresponding eigenstates, we go beyond simplified model systems. This allows us to study the influence of self-polarization and the response of the electronic structure to the cavity interaction on the spectral features even beyond the single-molecule case.

## I Introduction

Light–matter coupling between optical resonances of a cavity and molecular transitions can result in the formation of molecular polaritons.^1–4^ When coupling overcomes the dissipative processes, the strong coupling regime is reached, and these new hybridized states with mixed photon–matter character can be observed spectroscopically. A pair of characteristic peaks called the lower polariton (LP) and upper polariton (UP) can be observed shifted above and below the field-free transition frequency. By controlling the photonic environment, it becomes possible to selectively couple the cavity photon modes to vibrational or electronic transitions in molecules, called vibrational-strong coupling (VSC) and electronicstrong coupling (ESC), respectively. Both types of strong coupling are discussed as effective tools not only for modifying photophysics and photochemistry^5–8^ but also for altering electronic ground-state reaction rates and product branching ratios.^9–11^ However, the current theoretical description of VSC in particular is far from complete, and many open questions remain. For example, it is not clear how the polariton states contribute to the experimentally observed modification of reaction rates. The formation of polaritons in an ensemble is inherently a collective phenomenon in which the excitation is delocalized over the whole system. However, a chemical reaction is thought to occur locally in individual molecules.

Consequently, both linear^12–14^ and non-linear spectroscopy^15–22^ are methods that can be used to advance the understanding of molecular polaritonics. In particular, coherent multidimensional infrared spectroscopy is a powerful tool that can probe anharmonicities and vibrational energy relaxation pathways, opening the possibility of further insight into processes under VSC. For example, the off-diagonal peaks in 2D spectroscopy allow direct observation of the coherent energy exchange between the LP and UP states and can provide further insight into the kinetics and optical response of strongly coupled systems. In addition, the time-resolved spectra are also sensitive to the static and dynamical disorder that induces ultrafast polariton decay into the weakly coupled (dark) molecular states. Among the multi-pulse non-linear spectroscopic techniques available,^23^ we focus on the double quantum coherence (DQC) technique^22,24–27^ in this work. The use of DQC spectroscopy for molecular vibrational polaritons in optical cavities allows one to directly probe the double excitation manifold without being convolved with single exciton resonances. Therefore, the effect of strong couplings on the vibrational anharmonicities in the systems can be observed. In addition, in the context of electronic spectroscopy of molecular systems, the DQC signal is a direct measure of the electron correlation.^28^ Therefore, the DQC signal for vibrational polaritons provides information on the polariton correlation across interacting molecules.

In this paper, we simulate DQC spectra for a single HF molecule and a pair of HF molecules resonantly coupled to a single-photon mode of an optical cavity in the infrared. Our simulation is based on full-dimensional cavity potential energy surfaces (cPESs) calculated on the cavity Born–Oppenheimer Hartree–Fock (CBO-HF)^29,30^ level of theory. The CBO-HF ansatz allows the molecular system, i.e., the electronic structure, to respond to the cavity field due to the self-consistent field (SCF) procedure, and the inclusion of dipole self-energy (DSE) terms allows self-polarization^29–32^ of the coupled cavity–molecular system. In addition to a detailed analysis of the obtained DQC spectra, we also investigated the influence of the DSE, i.e., self-polarization, and the SCF procedure, i.e., the response of the electronic structure, on the spectral features. Finally, we study the changes introduced by going beyond a single molecule and the possible influence of cavity-mediated intermolecular interactions on the DQC signal.

## II Theory and Models

### A Cavity Born–Oppenheimer approximation

In our recent work,^29,33^ we have introduced the CBO-HF approach, which represents a formulation of the well-known Hartree–Fock ansatz in the context of the cavity Born–Oppenheimer approximation (CBOA). Within CBOA, the cavity field modes are grouped with the nuclei in a generalized Born–Huang expansion.^34,35^ This separation allows us to first solve the quantum problem of the electrons for a fixed nuclear and photonic configuration. Subsequently, the combined nuclear–photonic problem is solved fully quantum mechanically on the obtained cPES. The electronic CBOA Hamiltonian for a single cavity field mode takes the form of

(1) $${\hat{H}}_{CBO}={\hat{H}}_{el}+\frac{1}{2}\omega_{c}^{2}q_{c}^{2}-\omega_{c}q_{c}\left(\lambda_{c}\cdot \hat{\mu }\right)+\frac{1}{2}{\left(\lambda_{c}\cdot \hat{\mu }\right)}^{2},$$

where $\hat{\mu }$ represents the molecular dipole operator and *Ĥ*_*el*_ is the Hamiltonian for the field-free many-electron system. The second term is a harmonic potential introduced by the photon displacement field, with the photon displacement coordinate *q*_*c*_ as a parameter and *ω*_*c*_ being the frequency of the cavity mode. The third term describes the dipole coupling between the molecular system and the photon displacement field, which is characterized by the coupling strength ***λ***_*c*_. The last term is the DSE operator,^31,32,36^ which describes the self-polarization of the molecule–cavity system. The cavity mode-specific coupling parameter ***λ***_*c*_ for a cavity with effective mode volume *V*_*c*_ is defined as follows:

(2) $$\lambda_{c}=e_{c}\lambda_{c}=e_{c}\sqrt{\frac{4\pi }{V_{c}}}.$$

The unit vector ***e***_*c*_ denotes the polarization axis of the cavity mode.

Using the CBO-HF approach,^29,33^ we represent the many-electron wave function as a single Slater determinant formed from a set of orthonormal spin-orbitals. In this work, we also use two variations of the electronic CBOA Hamiltonian *Ĥ*_*CBO*_, describing the many-electron problem. In the first case labeled linear CBO-HF, we neglected the DSE operator and the resulting energy $E_{CBO}^{lin}$ reads

(3) $$\langle E_{CBO}^{lin}\rangle =E_{el}+E_{lin\;}+E_{dis\;}\;\;\text{with}\;\;E_{dis\;}=\frac{1}{2}\omega_{c}^{2}q_{c}^{2}.$$

For the second case, the standard CBO-HF Hamiltonian, including the DSE operator, is used,

(4) $$\langle E_{CBO}\rangle =E_{el}+E_{lin}+E_{dse}+E_{dis}.$$

As in the standard Hartree–Fock approach, in both cases the CBO-HF energy expectation values ⟨*E*_*CBO*_⟩ and $\langle E_{CBO}^{lin}\rangle$ for a given nuclear configuration and photonic displacement field can be determined using the self-consistent field method. We then use the converged CBO-HF wave function to determine the expectation value of the permanent dipole moment.

In addition to considering the full DSE operator, the SCF treatment itself has proved crucial for capturing relevant aspects in the description of strongly coupled molecules.^29,30,33^ To study this aspect, we used the field-free Hartree–Fock energy and expectation values of the dipole moment and the DSE operator to construct an extended Tavis–Cummings (ETC) model.^37–41^ The corresponding energy expectation value that defines the cPESs has the following form:

(5) $$\langle E_{ETC}\rangle =E_{el}-\omega_{c}q_{c}\langle \lambda_{c}\cdot {\hat{\mu }}_{0}\rangle +\frac{1}{2}{\langle {\left(\lambda_{c}\cdot \hat{\mu }\right)}^{2}\rangle }_{0}+\frac{1}{2}\omega_{c}^{2}q_{c}^{2}.$$

Both the linear light–matter interaction term and a quadratic DSE term are calculated with the corresponding field-free expectation values ${\langle \lambda_{c}\cdot \hat{\mu }\rangle }_{0}$ and ${\langle {\left(\lambda_{c}\cdot \hat{\mu }\right)}^{2}\rangle }_{0}$, respectively. Note that the main difference between the CBO-HF and the ETC approach is the optimization of the electronic wave function: in both versions of the CBO-HF calculation, the wave function is optimized in the presence of the electric field mode, while in the ETC model, the electronic wave function is optimized without the electric field mode.

### B Double quantum coherence spectroscopy

**Fig. 1:**
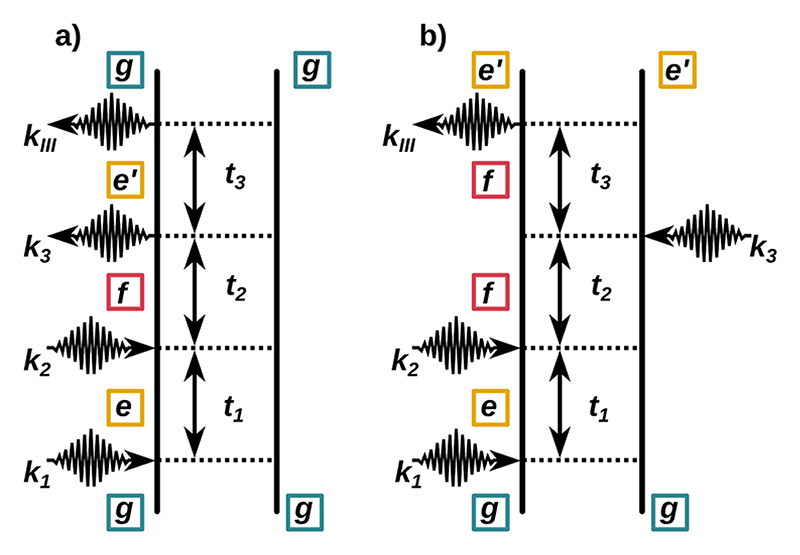
Two double sided Feynman diagrams that contribute to the DQC signal 𝒮(*t*_3_, *t*_2_, *t*_1_) in the phase-matching direction (***k***_*III*_ = ***k***_1_ + ***k***_2_ − ***k***_3_) in the rotating wave approximation. The ground state is colored green, the single excited states yellow, and the double excited states are red. The signal resulting from (a) and (b) correlates the double quantum excitation Ω_*gf*_ with the fundamental Ω_*e′g*_ and the hot band Ω_*fe*′_, respectively.

The DQC technique is a 2D-IR method performed with four temporally well-separated laser pulses. The first three pulses with wavevectors ***k***_1_, ***k***_2_, and ***k***_3_ generate a non-linear polarization in the molecular ensemble, which is heterodyne detected with the fourth pulse. We focus on the signal generated along the phase matching direction ***k***_*III*_ = ***k***_1_ + ***k***_2_ − ***k***_3_.^23,26,27^ The signal recorded against the three delay times between the pulses *t*_1_, *t*_2_, and *t*_3_ is denoted by 𝒮(*t*_3_, *t*_2_, *t*_1_). By invoking the rotating wave approximation, only the dominant contributions to 𝒮, where all interactions are resonant, are retained. Consequently, there are only two contributions to the ***k***_*III*_ signal for our coupled cavity molecule systems represented by the double-sided Feynman diagrams shown in Fig. 1. The two diagrams represent the same evolution during the first two time intervals: during *t*_1_ the density matrix oscillates with the frequency Ω_*eg*_ = *ϵ*_*e*_ − *ϵ*_*g*_, and during *t*_2_ the density matrix oscillates with the frequency Ω_*fg*_ = *ϵ*_*f*_ − *ϵ*_*g*_. Here, *ϵ*_*g*_, *ϵ*_*e*_, and *ϵ*_*f*_ are the vibrational/polaritonic eigenenergies of the vibrational/polaritonic ground state *g* (green), the first-excitation manifold *e* (yellow), and the second-excitation manifold *f* (red), respectively. During *t*_3_ the diagrams yield an oscillation frequency of either Ω_*e′g*_ or Ω_*fe*′_ [see Figs. 1(a) and 1(b), respectively]. Thus, transitions that form a harmonic ladder, that is, Ω_*e′g*_ = Ω_*fe′*_, cancel, and such transitions vanish in the DQC signal. Consequently, one of the main features of the DQC technique is its sensitivity to anharmonicities in the molecular systems studied or, in our case, polaritonic systems. Another one is the possibility of having direct access to information on not only the first but also the second excitation manifold. The obtained 3D signal 𝒮(*t*_3_, *t*_2_, *t*_1_) can be written as a double Fourier transform with respect to *t*_3_ and *t*_2_,

(6) $$S\left(\Omega_{3},\Omega_{2},t_{1}\right)=\int_{0}^{\infty }\int_{0}^{\infty }dt_{3}dt_{2}e^{i\left(\Omega_{3}t_{3}+\Omega_{2}t_{2}\right)}S\left(t_{3},t_{2},t_{1}\right).$$

Expanding the expression 𝒮(Ω_3_, Ω_2_, *t*_1_) in the basis of the polaritonic eigenstates, for *t*_1_ = 0, the signal becomes

(7) $$\begin{array}{l} S\left(\Omega_{3},\Omega_{2},t_{1}=0\right)=\underset{e,e^{\prime },f}{\sum }\frac{1}{\left(\Omega_{2}-\Omega_{fg}+i\gamma \right)}\\ \;\times \left[\frac{\mu_{ge^{\prime }}\mu_{e^{\prime }f}\mu_{fe}\mu_{eg}}{\left(\Omega_{3}-\Omega_{e^{\prime }g}+i\gamma \right)}-\frac{\mu_{ge^{\prime }}\mu_{e^{\prime }f}\mu_{fe}\mu_{eg}}{\left(\Omega_{3}-\Omega_{fe^{\prime }}+i\gamma \right)}\right], \end{array}$$

where *μ*_*ij*_ are the transition dipole moments between polaritonic states *i* → *j* and *γ* is an empirical dephasing rate. In this work, we assume an idealized case where a single value for *γ* is chosen for all three terms of Eq. (7).

As discussed in the literature,^42–45^ non-linear spectroscopy of molecules coupled to a cavity is not identical to the well-established situation of laser light and matter interacting in free space. Depending on the geometrical realization, both the pump and probe fields can interact directly with the coupled cavity field, leading to a non-linear interaction. We assume that an idealized infrared cavity would allow a measurement perpendicular to the cavity axis, i.e., the measurement is unaffected by the mirrors and avoids a non-linear interaction with the cavity field. Alternatively, this measurement can also be performed using four collinear pulses combined with the phase-cycling method in order to extract the signal originating from the appropriate Liouville pathways.

### C Computational details

The vibrational eigenenergies and the transition dipole moments needed to calculate the DQC spectra according to Eq. (7) are obtained using the CBO-HF ansatz in the Psi4NumPy environment,^46^ which is an extension of the PSI4^47^ electronic structure package. All calculations were performed using the aug-cc-pVDZ basis set,^48^ and the geometry of the isolated single HF molecule was optimized at the Hartree–Fock level of theory. In this study, we will consider the interaction between a lossless single mode cavity and an ensemble of *N*_*mol*_ = 1 and *N*_*mol*_ = 2 HF molecules. We assume that the molecules are aligned with the polarization of the cavity mode, and we consider a uniform electric field within the cavity. In order to compare the cases *N*_*mol*_ = 1 and *N*_*mol*_ = 2, we apply a scaling factor of $1/\sqrt{N_{mol}}$ on the collective coupling strength ***λ**_c_* to obtain a fixed Rabi splitting,

(8) $$\lambda_{c}=\frac{\lambda_{0}}{\sqrt{N_{mol}}}e_{c}.$$

Here, *λ*_0_ is equivalent to *λ*_*c*_ in Eq. (2) in the single-molecule case. As a result, we increase the mode volume *V*_*c*_ of the cavity, but by including more molecules, we keep the average density of molecules *N*_*mol*_ / *V*_*c*_ fixed. We use a coupling strength *λ*_0_ of 0.03 au, which corresponds to a cavity electric field strength of 1.5 V nm^−1^ in a Fabry–Pérot-like setup.

By scanning along the bond length of each HF molecule *r*_*HF*_ and the photon displacement coordinate *q*_*c*_, we construct the (*N*_*mol*_ + 1)-dimensional cPES together with the corresponding dipole moment surfaces. The potential energy surfaces for the cases *N*_*mol*_ = 1 and *N*_*mol*_ = 2 are interpolated to an equally spaced grid of 128 × 64 (*r*_*HF*_ × *q*_*c*_) grid points and 128 × 128 × 64 (*r*_*HF*_ × *r*_*HF*_ × *q*_*c*_) grid points, respectively. A Gaussian-shaped trial function is numerically propagated in imaginary time^49^ (time step 0.1 au and 70 000 time steps) on cPES with the Arnoldi propagation scheme^50^ to obtain the first ten nuclear–photonic eigenfunctions and to determine the corresponding transition dipole moments. All quantum dynamics simulations are performed with the open source quantum dynamics code QDng.^51^ All calculations were performed in a reproducible environment using the Nix package manager together with NixOS-QChem^52^ (commit f5dad404) and Nixpkgs (nixpkgs, 22.11, commit 594ef126).

## III Results

In the following, we present DQC spectra simulated for a single HF molecule and a pair of HF molecules resonantly coupled to a single-photon mode of an optical cavity. Self-consistent treatment within the CBO-HF ansatz allows the electronic structure of the molecular ensemble to respond to the cavity field. Moreover, the inclusion of DSE terms allows for a proper description of the self-polarization of the coupled cavity–molecular system.^29,30,32^

### A Vibrational polaritons in hydrogen fluoride molecules

**Fig. 2:**
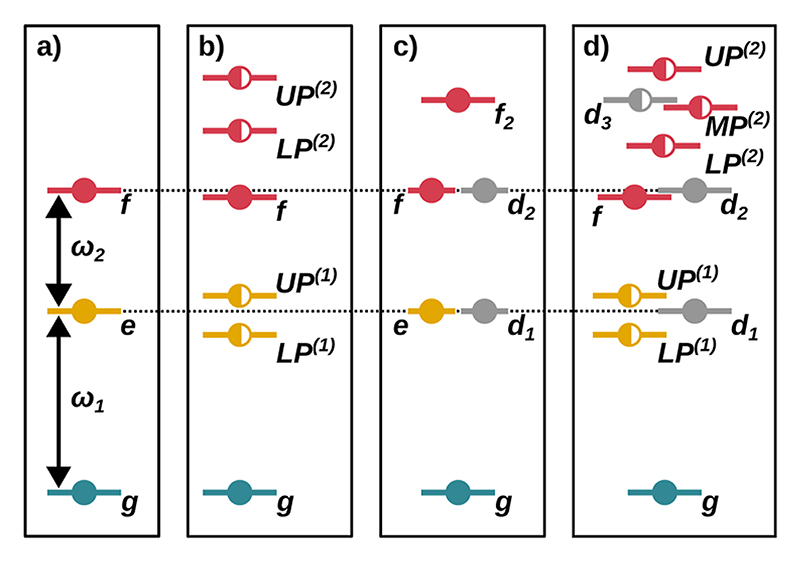
Schematic energy level diagrams for a single HF molecule and a pair of HF molecules without a cavity in (a) and (c) and resonantly coupled with a single cavity mode in (b) and (d). The cavity frequency *ω*_*c*_ is resonant with *ω*_1_. The ground state is colored green, the single excited state is colored yellow, and the double excited states are colored red. The optically dark states originating from the ground state to the single excited manifold and from the single excited manifold to the double excited manifold are shown in gray. Full circles indicate states of predominantly matter character, and half-filled circles indicate states with a mixed matter and photon contribution.

Figure 2 shows schematic energy level diagrams for a single HF molecule and two HF molecules without a cavity in (a) and (c) and resonantly coupled with a single cavity mode (*ω*_*c*_ = *ω*_1_) in (b) and (d), respectively. Apart from the expected energetic differences between the results obtained with the three different energy expectation values, the schematic energy-level diagrams shown are the same in all three cases. An analysis of the discussed polaritonic states for both the single-molecule case and the two-molecule case in terms of uncoupled bare states ∣*v, n*⟩ can be found in Sec. S1 of the supplementary material. In the notation used for the bare states, *v* describes the molecular vibrational excitation of the uncoupled system and *n* is the uncoupled photon number.

In the case of a single uncoupled HF molecule, the energy diagram shown in Fig. 2(a) consists of the vibrational ground state *g*, a single excited state *e*, and a double excited state *f*, which correspond to vibrational quantum numbers *v* = 0, 1, 2, respectively. These states are separated by *ω*_1_ = 4281 and *ω*_2_ = 4108 cm^−1^, respectively. The corresponding molecular anharmonicity Δ = *ω*_1_ − *ω*_2_ for HF is 173 cm^−1^. When a single-cavity photon mode strongly couples to a single HF molecule aligned with the cavity polarization axis, two single excited states and three double excited states in the system are formed; see Fig. 2(b). The cavity frequency *ω*_*c*_ is chosen to be resonant with the fundamental transition *ω*_1_ = 4281 cm^−1^, and the coupling strength *λ*_*c*_ is 0.03 au. A complementary analysis for the case *ω*_*c*_ = *ω*_2_, corresponding to the first hot transition, can be found in Sec. S2 of the supplementary material. Within the single excitation manifold, this resonant interaction leads to the expected formation of a pair of hybrid polaritonic states *UP*^(1)^ and *LP*^(1)^ with a Rabi splitting $\Omega_{R}^{\left(1\right)}$ of 60 cm^−1^ for the chosen coupling strength. The lowest energy state *f* in the second excitation manifold of the coupled cavity–molecule system is characterized mainly by a double excitation in the molecular part (*v* = 2, bare state ∣2, 0⟩). Compared to its cavity-free counterpart, this state is stabilized by ~20 cm^−1^. The remaining two states are on average Δ higher in energy than *f* and form a second pair of hybrid polaritonic states *UP*^(2)^ and *LP*^(2)^ with a Rabi splitting $\Omega_{R}^{\left(2\right)}$ of 90 cm^−1^. As expected, the Rabi splitting $\Omega_{R}^{\left(2\right)}$ in the second excitation manifold is increasing by approximately a factor of $\sqrt{2}$ compared to $\Omega_{R}^{\left(1\right)}$. *UP*^(2)^ and *LP*^(2)^ are formed by the bare state ∣1, 1⟩, in which the molecule and the cavity are both singly excited, and the bare state ∣0, 2⟩, in which the cavity is doubly excited. None of the states in this coupled cavity–molecule system is expected to be dark with respect to the ground state and the single excited manifold.

In the case of two HF molecules, in order to describe the bare molecular states, we use the basis ∣*v*_*s*_, *v*_*a*_⟩ formed by the two quantum numbers *v*_*s*_ and *v*_*a*_. The quantum numbers *v*_*s*_ and *v*_*a*_ correspond to the vibrational excitation of the symmetric and antisymmetric stretching normal modes, respectively. For more details, see Sec. S1 of the supplementary material. The two HF molecules are oriented parallel with respect to the cavity polarization axis and separated by 800 Å to ensure that the non-cavity-induced intermolecular couplings are negligible. Figure 2(c) shows the corresponding energy level diagram. The two single excited states *e* and *d*_1_ are energetically degenerate. Both correspond to the first excitation of the symmetric stretching mode (∣10⟩) and the antisymmetric stretching mode (∣01⟩) of the two HF molecules, respectively. This explains why the *e* state (symmetric mode) can be excited from the ground state, while the other state is dark because of symmetry. Similarly, the two doubly excited states *f* and *d*_2_ are energetically degenerate and correspond to the second excitation of the symmetric stretching mode and the antisymmetric stretching mode ∣20⟩ and ∣02⟩. Consequently, the *f* state is a bright state while *d*_2_ is dark. The remaining state *f*_2_ is Δ higher in energy and is formed by a simultaneous single excitation of both the symmetric and antisymmetric stretching mode ∣11⟩.

By resonantly coupling the two HF molecules to the single cavity mode, again a pair of hybrid polaritonic states *UP*^(1)^ and *LP*^(1)^ with a Rabi splitting $\Omega_{R}^{\left(1\right)}$ of 60 cm^−1^ is formed [see Fig. 2(d)]. Since the *d*_1_ state (antisymmetric stretching mode ∣01⟩) is dark, it is not affected by the coupling to the cavity. The double excitation manifold is more complex, with a total of six states. Both *f* (∣20⟩) and *d*_2_ (∣02⟩) are only slightly or not affected by the cavity interaction and do not change their predominantly molecular character. The four remaining states, *UP*^(2)^, *MP*^(2)^, *d*_3_, and *LP*^(2)^, all have a photonic contribution; see Table S2 in Sec. S1 of the supplementary material. However, only *UP*^(2)^, *MP*^(2)^, and *LP*^(2)^ are true hybrid polaritonic states, while the state *d*_3_ (∣01, 1⟩) is formed by adding a cavity excitation to the dark state *d*_1_ (∣01, 0⟩) without further hybridization. The splitting between the three polaritonic states is 53 cm^−1^, corresponding to a total Rabi splitting $\Omega_{R}^{\left(2\right)}$ of 106 cm^−1^. The two states *UP*^(2)^ and *LP*^(2)^ are mainly characterized by a hybridization of the two bare states ∣10, 1⟩ and ∣00, 2⟩. The middle polariton *MP*^(2)^ is mostly a linear combination of |00, 2⟩ and |11, 0⟩, which corresponds to *f*_2_ in the case of two uncoupled molecules [Fig. 2(c)].

### B DQC spectra for a single **HF** molecule

**Fig. 3:**
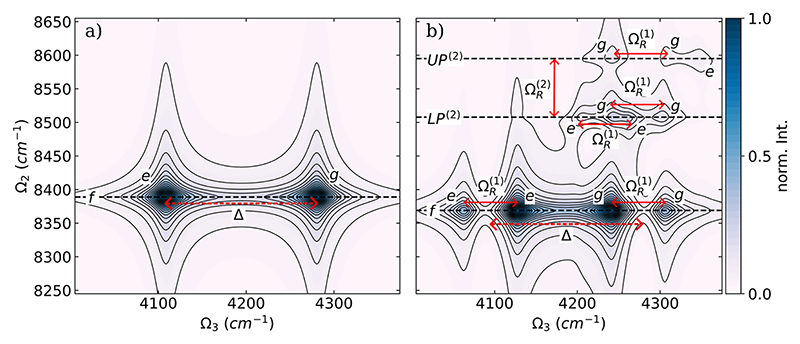
Absolute value of the normalized DQC spectra of a single HF molecule (a) without a cavity and (b) coupled to a single cavity mode with ω_c_ = 4281 cm^−1^. The coupling strength *λ*_*c*_ is 0.03 au, and the dephasing *γ* is 10 cm^−1^. The black horizontal dashed lines mark the energy of the final states, and all signals are labeled *e* and *g*, indicating that the initial state is the ground state or an intermediate state. The red lines with arrows highlight relevant energy differences. The signals were obtained using the full CBO-HF ansatz.

Figures 3(a) and 3(b) show the absolute value of the DQC spectra for a single HF molecule without a cavity and resonantly coupled with a single cavity mode, respectively. The normalized absolute values are used in the following to simplify the comparison between spectra obtained with different cPES and numbers of molecules.

As illustrated in Fig. 2(a), the HF molecule without a cavity is a simple three-level system, and the resulting DQC spectra exhibit two peaks [see Fig. 3(a)]. Both peaks are resonant at 8393 cm^−1^ on the Ω_2_ axis and separated by the molecular anharmonicity Δ of 173 cm^−1^ along the Ω_3_ axis. The one at Ω_3_ = 4281 cm^−1^ is due to the *g* → *e* transition, and the signal at 4108 cm^−1^ corresponds to the *e* → *f* transition. For the coupled single-molecule single-cavity mode system, we observe three distinct resonances (horizontal dashed lines) on the Ω_2_ axis in the DQC spectra [see Fig. 3(b)]. At Ω_2_ = 8369 cm^−1^, corresponding to the final state *f*, we observe four peaks, which are also the most intense of the whole DQC spectra. These peaks form a pair of doublets, where the two doublets are separated by the molecular anharmonicity Δ of 173 cm^−1^. The two peaks of each doublet are separated by the Rabi splitting $\Omega_{R}^{\left(1\right)}$ of 60 cm^−1^. The doublet around Ω_3_ = 4290 cm^−1^ is due to the transition from the ground state to the pair of hybrid polaritonic states *UP*^(1)^ and *LP*^(1)^, and the other is due to the transition from these hybrid states to the *f* state. Comparing the DQC spectra shown in Figs. 3(a) and 3(b), the stabilization of the state *f* due to the cavity interaction is clearly visible. The other two resonances on the Ω_2_ axis associated with the two hybrid states *UP*^(2)^ and *LP*^(2)^ are weaker and separated by a Rabi splitting $\Omega_{R}^{\left(2\right)}$ of 90 cm^−1^. At Ω_2_ = 8507 cm^−1^, final state *LP*^(2)^, four peaks are distinguishable at 4206 cm^−1^ (*LP*^(1)^ → *LP*^(2)^), at 4245 cm^−1^ (g → *LP*^(1)^), at 4260 cm^−1^ (*UP*^(1)^ → *LP*^(2)^), and at 4303 cm^−1^ (*g* → *UP*^(1)^) for the chosen dephasing. By careful analysis of the underlying transitions, these signals can be grouped into two doublets, with each peak approximately separated by the Rabi splitting $\Omega_{R}^{\left(1\right)}$ of 60 cm^−1^. For the final state *UP*^(2)^, Ω_2_ = 8595 cm^−1^, only three very weak signals can be observed at 4242 cm^−1^ (*g* → *LP*^(1)^), at 4305 cm^−1^ (*g* → *UP* ^(1)^), and at 4348 cm^−1^ (*LP*^(1)^ → *UP*^(2)^). The intensity of the fourth possible transition (*UP*^(1)^ → *UP*^(2)^) is too weak to be seen in the spectra. The first two peaks are again approximately split by the Rabi splitting $\Omega_{R}^{\left(1\right)}$.

**Fig. 4:**
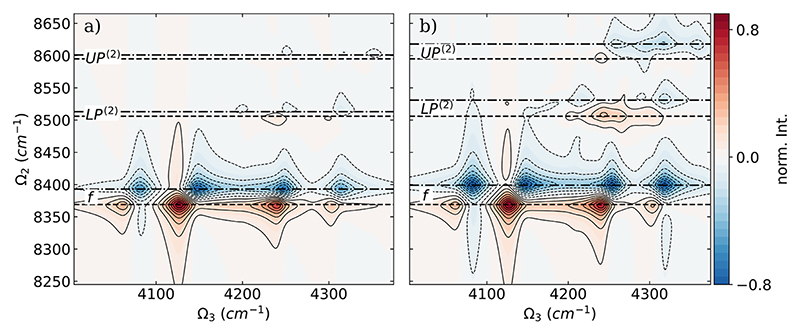
Difference of the DQC signal Δ 𝒮 of a single HF molecule coupled to a photon mode with *ω*_c_ = 4281 cm^−1^ between (a) full CBO-HF and linear CBO-HF and (b) full CBO-HF and ETC. Individual DQC spectra are normalized, and the absolute value is used to calculate the difference according to Eq. (9). The coupling strength *λ*_*c*_ is 0.03 au for both frequencies, and the dephasing *γ* is 10 cm^−1^. The energies of the final states are marked with black dashed lines for the full CBO-HF case and with dashed dotted lines for the linear CBO-HF and ETC cases, respectively.

Comparisons between DQC signals based on the full CBO-HF surface, the linear CBO-HF surface, and the ETC surfaces are shown as difference spectra Δ𝒮 in Fig. 4. Δ𝒮 is calculated using the normalized absolute value of the signals according to the following equation:

(9) $$\Delta S_{lin\;}={\left|S_{CBO}\right|}_{\text{norm}\;}-{\left|S_{CBO}^{lin}\right|}_{\text{norm}\;}$$

and

$$\Delta S_{ETC}={\left|S_{CBO}\right|}_{\text{norm}\;}-{\left|S_{ETC}\right|}_{\text{norm}}.$$

In both difference spectra, the peaks corresponding to the final state *f* are the most affected. All peaks associated with the *f* state are red-shifted by ~20–30 cm^−1^ in Ω_2_ and ~10–20 cm^−1^ in Ω_3_ when the DSE contribution is included [see Fig. 4(a)] or the SCF procedure is performed [see Fig. 4(b)]. As shown in Fig. 4(a), the *UP*^(2)^ resonance and the *LP*^(2)^ resonance and the corresponding peaks are almost unaffected when the DSE contribution is not included. In contrast, when the ETC surface is used to determine the DQC spectra, the resonances of *UP*^(2)^ and the resonances of *LP*^(2)^ are affected; see Fig. 4(b). Both are blue shifted by ~ 20 cm^−1^ in Ω_2_ for the case without SCF. In addition, the intensity distribution changes; *UP*^(2)^ loses intensity while *LP*^(2)^ gains it when going from the ETC spectra to the full CBO-HF results.

For completeness, the real (Re) and imaginary (Im) parts of the DQC spectra of the coupled single-molecule single-cavity mode system are shown in Fig. S6, Sec. S3 of the supplementary material.

### C DQC spectra for a pair of **HF** molecules

**Fig. 5:**
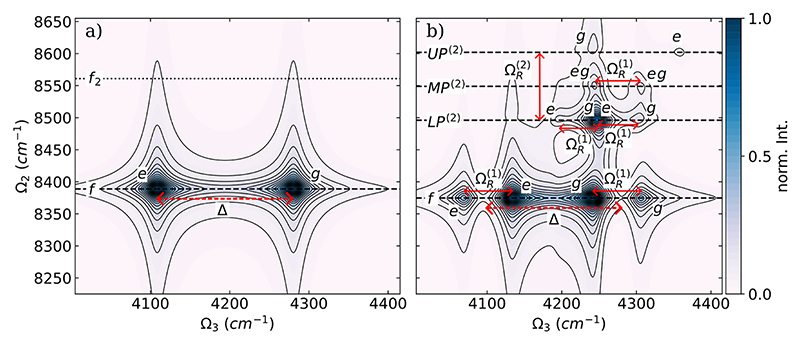
Absolute value of the normalized DQC spectra of two parallel oriented HF molecules (a) without a cavity and (b) coupled to a single cavity mode with *ω*_*c*_ = 4281 cm^−1^. The coupling strength *λ*_0_ is 0.03 au, and the dephasing *γ* is 10 cm^−1^. The black horizontal dashed lines mark the energy of the final states, and all signals are labeled *e* and *g*, indicating that the initial state is the ground state or an intermediate state. The red lines with arrows highlight relevant energy differences. The signals were obtained using the full CBO-HF ansatz.

The absolute value of the normalized DQC spectra for two identical HF molecules is shown in Fig. 5 for the case without a cavity in (a) and resonantly coupled with a single cavity mode in (b).

The DQC spectra of two parallel oriented HF molecules not interacting with a cavity field are shown in Fig. 5(a) and are nearly identical to the single-molecule situation; see Fig. 3(a) for comparison. The bright state *f*_2_, which is described by a simultaneous single excitation of both the symmetric and antisymmetric stretching mode, is not visible because of an exact cancellation of the two Liouville paths. The corresponding energy splittings between the *g* → *e* transition and the *e* → *f*_2_ transition are identical, leading to a harmonic ladder that is not visible in a DQC spectrum. A black dotted line in Fig. 5(a) indicates the energetic position of the corresponding hypothetical *f*_2_ resonances. The two additional vibrational states (*d*_1_ and *d*_2_) present in the case of two molecules are dark, as illustrated in Fig. 2(c), since they are associated with excitations of the antisymmetric linear combination of the two stretching modes. As a consequence, the resulting DQC spectrum has only two peaks and, thus, represents an effective three-level system. However, once the two HF molecules are resonantly coupled to the cavity mode, the resulting DQC spectrum, shown in Fig. 5(b), is clearly distinguishable from the single molecule case. We observe four distinct resonances (horizontal dashed lines) on the Ω_2_ axis in the DQC spectra since the other two possible double excited states *d*_2_ and *d*_3_ are dark for symmetry reasons. Similarly to the case of a single HF molecule, we observe four peaks at Ω_2_ = 8374 cm^−1^, corresponding to the final state *f*, which are also the most intense of the whole DQC spectrum. Interestingly, the resonance of the *f* state is nearly identical in both the one-molecule and two-molecule cases. The four associated peaks can again be grouped as a pair of doublets separated by the molecular anharmonicity Δ of about 173 cm^−1^ and the Rabi splitting $\Omega_{R}^{\left(1\right)}$ of 65 cm^−1^. Due to the rescaling of the coupling strength according to Eq. (8) $\Omega_{R}^{\left(1\right)}$ is similar to the one-molecule case. The doublet around Ω_3_ = 4274 cm^−1^ is due to the transition from the ground state to the pair of hybrid polaritonic states *UP*^(1)^ and *LP*^(1)^, and the other one is due to the transition from these hybrid states to the *f* state. The three hybrid polaritonic states *UP*^(2)^, *MP*^(2)^, and *LP*^(2)^ have visible resonances on the Ω_2_ axis. These three resonances are separated by about 53 cm^−1^, corresponding to a total Rabi splitting $\Omega_{R}^{\left(2\right)}$ of 106 cm^−1^, consistent with the observed increase of the Rabi splitting in the single molecule case. At Ω_2_ = 8496 cm^−1^, final state *LP*^(2)^, we see an intense peak at 4248 cm^−1^ comparable to the peaks corresponding to the final state *f* and two weaker signals at 4197 cm^−1^ (*LP*^(1)^ → *LP*^(2)^) and at 4303 cm^−1^ (*g* → *UP*^(1)^). These three signals are approximately separated by the Rabi splitting $\Omega_{R}^{\left(1\right)}$. The high intensity of the central peak can be explained by the overlap of the *g* → *LP*^(1)^ and *UP*^(1)^ →*LP*^(2)^ transitions. For the final state *MP*^(2)^, Ω_2_ = 8551 cm^−1^, only two signals can be observed at 4244 and at 4306 cm^−1^, separated again by the Rabi splitting $\Omega_{R}^{\left(1\right)}$. These two signals are formed by the transitions *g* → *LP*^(1)^ and *LP*^(1)^ → *MP*^(2)^ and the transitions *UP*^(1)^ → *MP*^(2)^ and *g* → *UP*^(1)^, respectively. As mentioned in Sec. III A, the final state *MP*^(2)^ is formed by hybridization of the two bare states |00, 2⟩ and |11, 0⟩. Without coupling to a cavity, this ∣11, 0⟩ state, labeled *f*_2_ in Fig. 5(a), is not visible because of a cancellation of the Liouville diagrams. In the cavity, however, this state becomes visible due to the hybridization, which induces anharmonicity in the corresponding excitation ladder. The identical process is observed when the cavity is resonant with the first hot transition and discussed in Sec. S2 of the supplementary material. Similarly to the single-molecule case, only two very weak signals corresponding to the final state *UP*^(2)^, Ω_2_ = 8610 cm^−1^, can be observed at 4242 cm^−1^ (*g* → *LP*^(1)^) and at 4359 cm^−1^ (*UP*^(1)^ → *UP*^(2)^). All other possible transitions are too weak to be observed in the spectra. There is no clear indication that any of the three dark states, *d*_1_ and *d*_2_, take part in the signal, and thus, these states are not needed to explain the DQC signals. As for the single molecule case, for completeness, we show the real (Re) and imaginary (Im) parts of the DQC spectra of the two-molecule case coupled to a single-cavity mode in Fig. S7, Sec. S3 of the supplementary material.

**Fig. 6:**
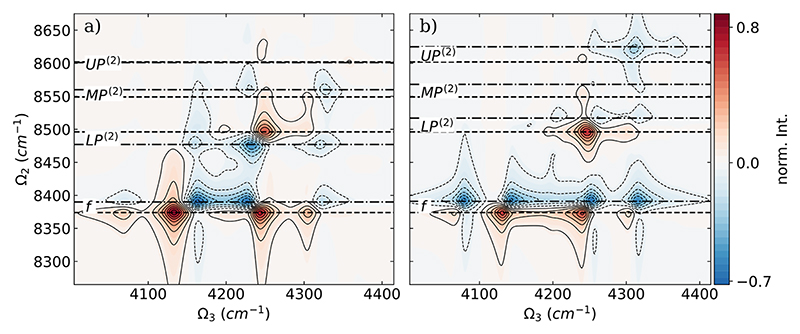
Difference Δ𝒮 of the DQC signal of two parallel HF molecules coupled to a photon mode with *ω*_*c*_ = 4281 cm^−1^ between (a) full CBO-HF and linear CBO-HF and (b) full CBO-HF and ETC. Individual DQC spectra are normalized, and the absolute value is used to calculate the difference according to Eq. (9). The coupling strength *λ*_*c*_ is 0.03 au for both frequencies, and the dephasing *γ* is 10 cm^−1^. The energies of the final states are marked with black dashed lines for the full CBO-HF case and with dashed dotted lines for the linear CBO-HF and ETC cases, respectively.

In Fig. 6, the comparison between the DQC signals based on the full CBO-HF surface, the linear CBO-HF surface, and the ETC surface for the two-molecule case is shown as difference spectra Δ*S* calculated according to Eq. (9). In agreement with the single-molecule case, the peaks corresponding to the state *f* are most affected in both difference spectra. For the comparison between the full CBO-HF and linear CBO-HF results shown in Fig. 6(a), the *f* peaks are only red-shifted by about 20 cm^−1^ in Ω_2_, while no general trend for shifts in Ω_3_ is apparent. When the DSE term is not included [see Fig. 6(a)], the resonances of the two hybrid polaritonic states *MP*^(2)^ and *LP*^(2)^ are affected. In particular, for the *LP*^(2)^ state, a red shift is observed in both Ω_2_ and Ω_3_ for the strong signal around 4248 cm^−1^ without DSE included. Interestingly, the weak *UP*^(2)^ remains almost unchanged, similar to the case of a single molecule. In contrast, when the ETC surface is used to determine the DQC spectra, the observed changes in the difference spectra in the single-molecule case and the two-molecule case are quite similar. For the *f* signals, almost the same red shift is observed in both Ω_2_ and Ω_3_; compare Figs. 4(b) and 6(b). The resonances of three hybrid polaritonic states, *UP*^(2)^, *MP*^(2)^, and *LP*^(2)^, are all blue shifted by roughly 20 cm^−1^ in Ω_2_ for the ETC case. Consistent with the single-molecule results, the intensity distribution changes; *UP*^(2)^ loses intensity while *LP*^(2)^ gains it when going from the ETC spectra to the full CBO-HF results. In general, the observed effect on the DQC spectra of the SCF treatment is the same for one and two HF molecules, where the DSE included in the CBO-HF approach shows additional changes in the spectra when going from one to two molecules.

**Fig. 7:**
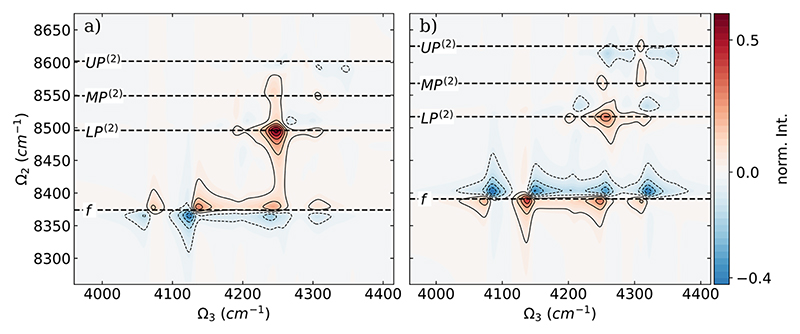
Difference of the absolute values of the DQC spectra of two parallel oriented HF molecules and a single HF molecule coupled to a photon mode with *ω*_0_ = 4281 cm^−1^, λ_*c*_ = 0.03 au, and the dephasing *γ* is 10 cm^−1^. (a) The full CBO-HF cPESs are used to construct the DQC spectra, and (b) the ETC model cPESs are used. The energies of the final states are marked with black dashed lines for the two molecule case.

As a final step, we want to further analyze the differences in the DQC spectra when going from one to two HF molecules for the full CBO-HF approach and using the ETC model. The corresponding difference spectra are shown in Fig. 7. Analyzing the difference spectrum in Fig. 7(a) for the full CBO-HF case, two main changes appear when going from one to two HF molecules. The most striking change is the increase in intensity for the signal around 4248 cm^−1^, which corresponds to the final state *LP*^(2)^. As discussed for the two-molecule DQC spectrum shown in Fig. 5(b), this intense peak is due to two overlapping signals that are still separated in the single-molecule DQC spectrum [see Fig. 3(b)]. The other change is a blue shift in Ω_2_ of all signals corresponding to the *f* resonance. For the ETC case shown in Fig. 7(b), all peaks associated with the final state *f* are shifted to lower frequencies in both Ω_2_ and Ω_3_. With respect to polaritonic states, only smaller changes are observed when going from one to two HF molecules. The distinct differences in the comparison between the full CBO-HF results and those obtained using the ETC model are due solely to the SCF process, which allows the electronic structure to respond to the cavity field mode. This response seems to be particularly relevant when going beyond a single-molecule situation because of cavity-induced molecular interactions.

## IV Summary and Conclusion

Based on the recently formulated cavity Born–Oppenheimer Hartree–Fock ansatz^29,33^ and inspired by the work of Saurabh and Mukamel,^27^ we simulated two-dimensional DQC spectra for the rather anharmonic case of one and two diatomic hydrogen fluoride HF molecules coupled to an infrared cavity. In both cases, the molecular system is coupled to a single-photon mode that is resonant with the first vibrational transition in HF. Using *ab initio* cPES at the CBO-HF level of theory, we could demonstrate how groundstate vibrational excitation manifolds are modified upon coupling to a cavity mode. As a consequence, the molecular anharmonicities, the molecule–cavity interaction, and the self-polarization of the molecule–cavity system are naturally included. Even in the rather simple diatomic case of HF, coupling to an optical cavity results in a rather complex and “asymmetric” DQC signal compared to model systems reported in the literature.^27^ This complexity comes from the non-idealized second excitation manifold, where, due to the anharmonicity of HF, both nearly pure molecular states and the expected hybrid polaritonic states contribute, as shown in Fig. 2. The significantly different character of these states leads to a mixture of rather weak and rather strong transitions. We were able to show that neglecting the DSE contribution or not performing the SCF procedure has a significant effect on the DQC signals. In both cases, the resonances of the *f* state are affected, which is mainly an excited molecular final state. Therefore, it allows us to monitor the effect of DSE and SCF on the LP and UP states in the single excitation manifold. However, for the single-molecule case without SCF, the *UP*^(2)^ state and the *LP*^(2)^ state also change. For the case of two HF molecules, the DSE contribution not only influences the *f* resonances but also leads to a strong signal corresponding to the final state *LP*^(2)^, which is clearly weaker without DSE or SCF. This *LP*^(2)^ signal is also the striking difference when going from a single molecule to a pair of molecules. In contrast, the resonances of the *f* state are nearly identical as the number of molecules increases.

An important result of our study is that we show that the DQC spectrum is highly sensitive to the effect of the DSE. In particular, when considering the case of two molecules without a cavity, the molecular state |11❳ corresponding to two simultaneously excited molecules does not contribute to the DQC spectrum due to the fact that the two molecules do not interact. However, when considering two molecules inside the cavity, the cavity induces an effective interaction between the two molecules resulting from the DSE, which can be directly probed by the DQC spectrum through the MP^(2)^ state. Therefore, we show that the DQC technique offers a unique insight into the cavity-mediated intermolecular interactions. DQC spectroscopy can be used to have a better understanding of many-body effects in molecular systems under vibrational strong coupling, which in turn may thus provide a deeper mechanistic insight into chemical reactions under vibrational strong coupling.

## Supplementary Material

Supplementary Material

## Data Availability

The data that support the findings of this study are available from the corresponding author upon reasonable request.
