# Supplementary material for: Disentangling collective coupling in vibrational polaritons with double quantum coherence spectroscopy
Source: J Chem Phys. Author manuscript; Available in PMC 2025 Jan 21. (PMC7617315; doi:10.1063/5.0239877)
Supplement: Supplementary Material [file EMS202212-supplement-Supplementary_Material.pdf]

Supporting Information:

Disentangling collective coupling in vibrational polaritons with double quantum coherence spectroscopy

Thomas Schnappinger,[1, a)] Cyril Falvo,[2, 3] and Markus Kowalewski[1, b)]

[1)] *Department of Physics, Stockholm University, AlbaNova University Center, SE-106 91 Stockholm, Sweden*

[2)] *Université Paris-Saclay, CNRS, Institut des Sciences Moléculaires d'Orsay, 91405 Orsay, France*

[3)] *Université Grenoble-Alpes, CNRS, LIPhy, 38000 Grenoble, France*

(Dated: 15 November 2024)

[a)]Electronic mail: thomas.schnappinger@fysik.su.se

[b)]Electronic mail: markus.kowalewski@fysik.su.se

CONTENTS

S1. Analysis of the vibrational polaritonic eigenstates 3

S2. DQC spectra for a cavity resonant with the first hot transition of HF 9

S3. Real and imaginary parts of the DQC spectra 14

S1. ANALYSIS OF THE VIBRATIONAL POLARITONIC EIGENSTATES

To analyze the polaritonic eigenstates for a single HF molecule and a two HF molecules coupled to a single-photon mode of an optical cavity, we extended the corresponding coupled eigenfunctions χ_j in terms of the uncoupled bare states:

$$|\chi_j\rangle = \sum_i c_{i,j} |v, n\rangle_i \tag{S1}$$

where v describes the vibrational excitation of the uncoupled molecular system and n is the photon number. The molecular part v of the uncoupled molecular system is qualitatively described in terms of normal modes. In the case of two HF molecules, the two included normal modes sketched in Fig. S1 of the ensemble are the symmetric linear combination and the antiymmetric linear combination of the individual molecular stretching modes. We formulate $|v\rangle$ for two HF molecules as $|v_s v_a\rangle$, where v_s and v_a are the excitation numbers in the symmetric and antisymmetric stretching modes, respectively. Consequently, two molecular ensemble states $|10\rangle$ and $|01\rangle$ exist in the first excitation manifold, representing the first excitation of the symmetric stretching mode and the antisymmetric stretching mode, respectively. The two states of the molecular ensemble $|20\rangle$ and $|02\rangle$ describe the corresponding second excitation in these stretching modes. The remaining double excited state $|11\rangle$ describes the simultaneous single excitation of the symmetric stretching mode and the antisymmetric stretching mode. Apart from the numerical differences between the results

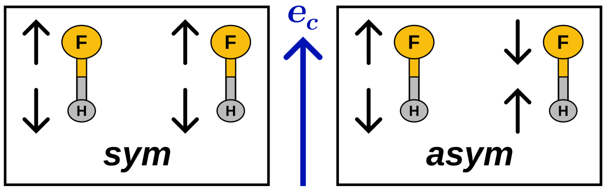

FIG. S1. Sketch of the symmetric (sym) and antiymmetric (asym) liner combinations for two HF molecules and the cavity polarization axis is shown in blue.

obtained with the three different energy expectation values, the character of the polaritonic states expressed in terms of the bare states is qualitatively the same in all three cases. Therefore, we will only show the results based on the full cavity Born-Oppenheimer Hartree-Fock (CBO-HF) energy expectation values. As a side note, we want to clarify that this basis

is not complete but sufficient to qualitatively describe the light-matter hybridization in the first and second excitation manifold.

The absolute squares of the coefficients $|c_{i,j}|^2$ of all polaritonic states discussed in the case of a single HF molecule coupled to a cavity with $\omega_c = \omega_1 = 4281\,\mathrm{cm}^{-1}$ and a coupling strength λ_c of 0.03 au are given in TABLE S1. The absolute squares of the coefficients $|c_{i,j}|^2$ of all polaritonic states discussed for the case of two HF molecules coupled to a cavity with$\omega_c = \omega_1 = 4281\,\mathrm{cm}^{-1}$ and a coupling strength λ_c of 0.03 au are given in are given in TABLE S3. In addition, the transition energies and transition dipole moments of all relevant vibropolaritonic states are given in TABLE S2 and TABLE S4. The corresponding coefficients $|c_{i,j}|^2$ of all polaritonic states discussed in section S2 for the case of one and two HF molecule and a cavity frequency $\omega_c = \omega_2 = 4108\,\mathrm{cm}^{-1}$ are given in TABLE S5 and TABLE S7. The transition energies and transition dipole moments of all relevant vibropolaritonic states are given in TABLE S6 and TABLE S8.

	$\|0,0\rangle$	$\|1,0\rangle$	$\|0,1\rangle$	$\|2,0\rangle$	$\|0,2\rangle$	$\|1,1\rangle$
$\|g\rangle$	**0.9945**	0.0055	0.0000	0.0000	0.0000	0.0000
$\|LP^{(1)}\rangle$	0.0030	**0.5301**	**0.4582**	0.0000	0.0059	0.0027
$\|UP^{(1)}\rangle$	0.0025	**0.4535**	**0.5359**	0.0000	0.0050	0.0031
$\|f\rangle$	0.0000	0.0000	0.0002	**0.9637**	0.0008	0.0287
$\|LP^{(2)}\rangle$	0.0000	0.0063	0.0024	0.0179	**0.5502**	**0.4088**
$\|UP^{(2)}\rangle$	0.0000	0.0046	0.0032	0.0121	**0.4216**	**0.5448**

TABLE S1. The absolute squares of the coefficients $|c_{i,j}|^2$ of all polaritonic states for the case of a single resonantly coupled HF molecule. The coefficients of the main contributions are highlighted in bold. The values are obtained using the full CBO-HF energy expectation values, a cavity frequency $\omega_c = \omega_1 = 4281\,\mathrm{cm}^{-1}$ and a coupling strength λ_c of 0.03 au.

	transition dipole $\hat{\mu}$			
	ω_{gx}	$\langle g\|$	$\langle LP^{(1)}\|$	$\langle UP^{(1)}\|$
$\|LP^{(1)}\rangle$	4242.60	0.0450	–	–
$\|UP^{(1)}\rangle$	4304.59	0.0275	0.0129	–
$\|f\rangle$	8368.56	0.0035	0.0593	0.0449
$\|LP^{(2)}\rangle$	8506.54	0.0019	0.0321	0.0351
$\|UP^{(2)}\rangle$	8592.46	0.0020	0.0180	0.0136

TABLE S2. All relevant transition energies and transition dipole moments of the vibropolaritonic states for the case of a single resonantly coupled HF molecule. The energies are given in cm^{-1} and the dipole moments in au. The values are obtained using the full CBO-HF energy expectation values, a cavity frequency $\omega_c = \omega_1 = 4281\,\mathrm{cm}^{-1}$ and a coupling strength λ_c of 0.03 au.

	$\|00,0\rangle$	$\|01,0\rangle$	$\|10,0\rangle$	$\|00,1\rangle$	$\|02,0\rangle$	$\|20,0\rangle$	$\|01,1\rangle$	$\|10,1\rangle$	$\|11,0\rangle$	$\|00,2\rangle$
$\|g\rangle$	**0.9890**	0.0000	0.0000	0.0109	0.0000	0.0000	0.0000	0.0000	0.0000	0.0000
$\|d_1\rangle$	0.0000	**0.9886**	0.0000	0.0000	0.0000	0.0000	0.0111	0.0002	0.0000	0.0000
$\|LP^{(1)}\rangle$	0.0060	0.0000	**0.4544**	**0.5224**	0.0000	0.0000	0.0000	0.0052	0.0000	0.0117
$\|UP^{(1)}\rangle$	0.0049	0.0000	**0.5341**	**0.4449**	0.0000	0.0000	0.0000	0.0059	0.0000	0.0099
$\|d_2\rangle$	0.0000	0.0001	0.0000	0.0000	**0.9740**	0.0000	0.0000	0.0138	0.0002	0.0000
$\|f\rangle$	0.0000	0.0000	0.0002	0.0000	0.0000	**0.9720**	0.0002	0.0002	0.0150	0.0004
$\|d_3\rangle$	0.0000	0.0111	0.0000	0.0000	0.0144	0.0000	**0.9374**	0.0147	0.0000	0.0000
$\|LP^{(2)}\rangle$	0.0000	0.0000	0.0053	0.0087	0.0000	0.0109	0.0069	**0.4396**	**0.1326**	**0.3710**
$\|MP^{(2)}\rangle$	0.0000	0.0000	0.0001	0.0071	0.0000	0.0001	0.0001	0.0071	**0.6566**	**0.3102**
$\|UP^{(2)}\rangle$	0.0000	0.0000	0.0057	0.0059	0.0000	0.0052	0.0077	**0.4896**	**0.1987**	**0.2643**

TABLE S3. The absolute squares of the coefficients $|c_{i,j}|^2$ of all polaritonic states for the case of two resonantly coupled HF molecules. The coefficients of the main contributions are highlighted in bold. The values are obtained using the full CBO-HF energy expectation values, a cavity frequency $\omega_c = \omega_1 = 4281\,\mathrm{cm}^{-1}$ and a coupling strength λ_c of 0.03 au.

	transition dipole $\hat{\mu}$				
	ω_{gx}	$\langle g\|$	$\langle LP^{(1)}\|$	$\langle d_1\|$	$\langle UP^{(1)}\|$
$\|LP^{(1)}\rangle$	4242.70	0.0636	–	–	–
$\|d_1\rangle$	4277.23	0.0000	0.0000	–	–
$\|UP^{(1)}\rangle$	4304.72	0.0364	0.0129	0.0000	–
$\|f\rangle$	8374.73	0.0053	0.0626	0.0000	0.0469
$\|d_2\rangle$	8375.34	0.0000	0.0000	0.0742	0.0000
$\|LP^{(2)}\rangle$	8496.39	0.0022	0.0678	0.0000	0.0242
$\|MP^{(2)}\rangle$	8549.80	0.0004	0.0310	0.0000	0.0500
$\|d_3\rangle$	8552.92	0.0000	0.0000	0.0107	0.0000
$\|UP^{(2)}\rangle$	8602.21	0.0019	0.0100	0.0000	0.0388

TABLE S4. All relevant transition energies and transition dipole moments of the vibropolaritonic states for the case of two resonantly coupled HF molecules. The energies are given in cm^{-1} and the dipole moments in au. The values are obtained using the full CBO-HF energy expectation values, a cavity frequency $\omega_c = \omega_1 = 4281\,\mathrm{cm}^{-1}$ and a coupling strength λ_c of 0.03 au.

	$\|0,0\rangle$	$\|0,1\rangle$	$\|1,0\rangle$	$\|0,2\rangle$	$\|1,1\rangle$	$\|2,0\rangle$
$\|g\rangle$	**0.9942**	0.0057	0.0000	0.0000	0.0000	0.0000
$\|p^{(1)}\rangle$	0.0057	**0.9706**	0.0124	0.0112	0.0000	0.0000
$\|e\rangle$	0.0000	0.0122	**0.9815**	0.0001	0.0060	0.0000
$\|p^{(2)}\rangle$	0.0000	0.0111	0.0002	**0.9463**	0.0248	0.0006
$\|LP^{(2)}\rangle$	0.0000	0.0002	0.0030	0.0162	**0.4649**	**0.5062**
$\|UP^{(2)}\rangle$	0.0000	0.0000	0.0030	0.0090	**0.4920**	**0.4866**

TABLE S5. The absolute squares of the coefficients $|c_{i,j}|^2$ of all polaritonic states for the case of a single resonantly coupled HF molecule. The coefficients of the main contributions are highlighted in bold. The values are obtained using the full CBO-HF energy expectation values, a cavity frequency $\omega_c = \omega_2 = 4108\,\mathrm{cm}^{-1}$ and a coupling strength λ_c of 0.03 au.

	transition dipole $\hat{\mu}$			
	ω_{gx}	$\langle g\|$	$\langle p^{(1)}\|$	$\langle e\|$
$\|p^{(1)}\rangle$	4092.78	0.0237	–	–
$\|e\rangle$	4281.86	0.0459	0.0060	–
$\|p^{(2)}\rangle$	8185.38	0.0001	0.0341	0.0022
$\|LP^{(2)}\rangle$	8339.74	0.0018	0.0381	0.0570
$\|UP^{(2)}\rangle$	8424.84	0.0041	0.0253	0.0420

TABLE S6. All relevant transition energies and transition dipole moments of the vibropolaritonic states for the case of a single resonantly coupled HF molecule. The energies are given in cm^{-1} and the dipole moments in au. The values are obtained using the full CBO-HF energy expectation values, a cavity frequency $\omega_c = \omega_2 = 4108\,\mathrm{cm}^{-1}$ and a coupling strength λ_c of 0.03 au.

	$\|00,0\rangle$	$\|00,1\rangle$	$\|01,0\rangle$	$\|10,0\rangle$	$\|00,2\rangle$	$\|01,1\rangle$	$\|10,1\rangle$	$\|02,0\rangle$	$\|20,0\rangle$	$\|11,0\rangle$
$\|g\rangle$	**0.9771**	0.0226	0.0000	0.0000	0.0003	0.0000	0.0000	0.0000	0.0000	0.0000
$\|p^{(1)}\rangle$	0.0222	**0.9104**	0.0000	0.0229	0.0431	0.0003	0.0003	0.0000	0.0000	0.0000
$\|d_1\rangle$	0.0000	0.0000	**0.9763**	0.0000	0.0000	0.0133	0.0101	0.0000	0.0000	0.0000
$\|e\rangle$	0.0004	0.0220	0.0000	**0.9534**	0.0010	0.0098	0.0129	0.0000	0.0000	0.0000
$\|p^{(2)}\rangle$	0.0002	0.0424	0.0000	0.0011	**0.8459**	0.0188	0.0248	0.0005	0.0006	0.0003
$\|d_2\rangle$	0.0000	0.0000	0.0132	0.0000	0.0000	**0.2910**	**0.2205**	**0.2430**	**0.1955**	0.0000
$\|LP^{(2)}\rangle$	0.0000	0.0013	0.0000	0.0115	0.0284	**0.1906**	**0.2516**	**0.2099**	**0.2609**	0.0092
$\|d_3\rangle$	0.0000	0.0000	0.0102	0.0000	0.0000	**0.2383**	**0.1805**	**0.2976**	**0.2395**	0.0000
$\|UP^{(2)}\rangle$	0.0000	0.0006	0.0000	0.0103	0.0146	**0.1811**	**0.2390**	**0.2243**	**0.2787**	0.0166
$\|f_2\rangle$	0.0000	0.0000	0.0000	0.0005	0.0003	0.0104	0.0137	0.0003	0.0004	**0.9495**

TABLE S7. The absolute squares of the coefficients $|c_{i,j}|^2$ of all polaritonic states for the case of two resonantly coupled HF molecules. The coefficients of the main contributions are highlighted in bold. The values are obtained using the full CBO-HF energy expectation values, a cavity frequency $\omega_c = \omega_2 = 4108\,\mathrm{cm}^{-1}$ and a coupling strength λ_c of 0.03 au.

		transition dipole $\hat{\mu}$			
	ω_{gx}	$\langle g|$	$\langle p^{(1)}|$	$\langle d_1|$	$\langle e|$
$|p^{(1)}\rangle$	4078.38	0.0464	–	–	–
$|d_1\rangle$	4273.42	0.0000	0.0000	–	–
$|e\rangle$	4282.75	0.0617	0.0080	0.0000	–
$|p^{(2)}\rangle$	8156.44	0.0003	0.0664	0.0000	0.0026
$|d_2\rangle$	8322.31	0.0000	0.0000	0.0722	0.0000
$|LP^{(2)}\rangle$	8330.31	0.0023	0.0514	0.0000	0.0713
$|d_3\rangle$	8411.48	0.0000	0.0000	0.0326	0.0000
$|UP^{(2)}\rangle$	8412.28	0.0058	0.0331	0.0000	0.0241
$|f_2\rangle$	8557.03	0.0009	0.0007	0.0000	0.0660

TABLE S8. All relevant transition energies and transition dipole moments of the vibropolaritonic states for the case of two resonantly coupled HF molecules. The energies are given in cm^{-1} and the dipole moments in au. The values are obtained using the full CBO-HF energy expectation values, a cavity frequency $\omega_c = \omega_1 = 4108\,\mathrm{cm}^{-1}$ and a coupling strength λ_c of 0.03 au.

S2. DQC SPECTRA FOR A CAVITY RESONANT WITH THE FIRST HOT TRANSITION OF HF

In this section, we briefly discuss the formed vibrational polaritons and the corresponding double quantum coherence (DQC) spectra when the cavity mode is resonant with the first hot transition of the HF molecule, $\omega_c = \omega_2 = 4108\,\mathrm{cm}^{-1}$.

Figure S2 shows the corresponding schematic energy level diagrams for a single HF molecule and a pair of HF molecules without a cavity in a) and c) and resonantly coupled with a single cavity mode in b) and d). Apart from the expected energetic differences between the results obtained with the three different energy expectation values, the schematic energy-level diagrams shown are the same in all three cases. A analysis of the discussed polaritonic states for both the single-molecule case and the two-molecule case in terms of uncoupled bare states $|k, n\rangle$ can be found in section S1 TABLE S5 and TABLE S7.

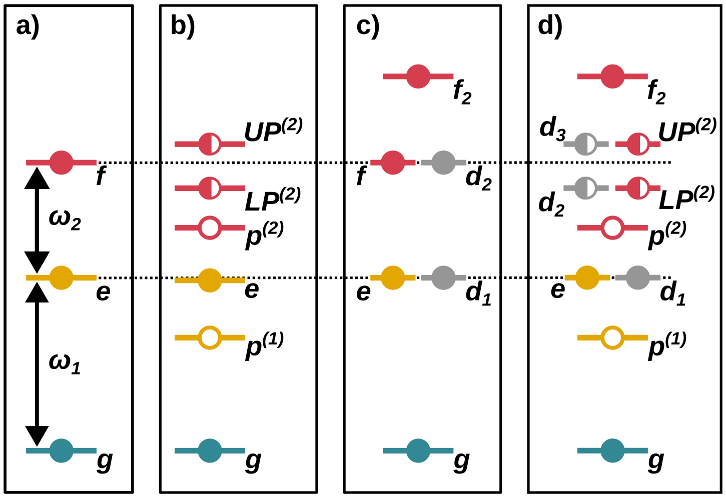

FIG. S2. Schematic energy level diagrams for a single HF molecule and a pair of HF molecules without a cavity in a) and c) and resonantly coupled with a single cavity mode in b) and d). The cavity frequency ω_c is resonant with ω_2. The ground state is colored green, the single excited state is colored yellow, and the double excited states are colored red. The optically dark states originating from the ground state to the single excited manifold and from the single excited manifold to the double excited manifold are shown in gray. Full circles indicate states of predominantly matter character, empty circles indicate states of predominantly photonic character, and half-filled circles indicate states with a mixed matter and photon contribution.

Since the uncoupled energy levels are discussed in the manuscript section III A, we restrict the discussion to the two cavity-coupled cases shown in Figure S2 b) and d). Within the first excitation manifold (color coded yellow), the cavity mode is out of resonance $\omega_c = \omega_1 - \Delta$ due to the anharmonicity of HF, and consequently the molecular state e (and d_1 in the case of two molecules) is almost unchanged compared to the field-free situation. However, because of the remaining weak off-resonance interaction, the photonic $p^{(1)}$ state (bare state $|0, 1\rangle$) still has a small molecular contribution.

The expected formation of the lower polariton (LP) state and the upper polariton (UP) state is observed only in the second excitation manifold, since the cavity is resonant with the first hot transition. As shown in Figure S2 b) Figure S2 d), the corresponding $UP^{(2)}$ state and the $LP^{(2)}$ state are separated in both cases by a Rabi splitting $\Omega_R^{(2)}$ of about $60\,\mathrm{cm}^{-1}$. In the case of a single HF molecule, these polaritonic states are formed by a linear combination of the two bare states $|1, 1\rangle$ and $|2, 0\rangle$. The remaining state $p^{(2)}$ is almost fully photonic (bare state $|0, 2\rangle$) and has only a small molecular contribution. For two HF molecules coupled to the cavity mode, in addition to the two polaritonic states $UP^{(2)}$ and $LP^{(2)}$, two energetically degenerate dark states d_2 and d_3 are present. These dark states are also formed by the four bare states $|01, 1\rangle$, $|10, 1\rangle$, $|02, 0\rangle$, and $|20, 0\rangle$ similarly to $UP^{(2)}$ and $LP^{(2)}$, but the leading components are excitations of the dark antisymmetric stretching modes ($|011\rangle$ and $|02, 1\rangle$). The remaining doubly excited state f_2 is Δ higher in energy than the average of the $UP^{(2)}$ state and the $LP^{(2)}$ state and is formed by a single simultaneous excitation of both stretching modes ($|11\rangle$).

The corresponding normalized absolute values of the DQC spectra for one HF molecule are shown in Figure S3. For the coupled single-molecule case shown in Figure S3 b), the main difference from situation $\omega_c = \omega_1$ is the presence of only two resonances on the Ω_2 axis associated with the two hybrid states $UP^{(2)}$ and $LP^{(2)}$ separated by the Rabi splitting $\Omega_R^{(2)}$. The peaks corresponding to the final state $LP^{(2)}$ are more intense, but for both resonances two main peaks and two weak side bands are observed. The main peaks are transitions into (around $4300\,\mathrm{cm}^{-1}$) and out of (around $4050\,\mathrm{cm}^{-1}$) the molecular state e, while the weak side bands are transitions involving the mostly photonic state $p^(1)$.

The corresponding normalized absolute values of the DQC spectra for two HF molecules are shown in Figure S4. The main difference in the DQC spectra for the two coupled HF molecules shown in Figure S4 b) compared to the case of a single molecule is the presence

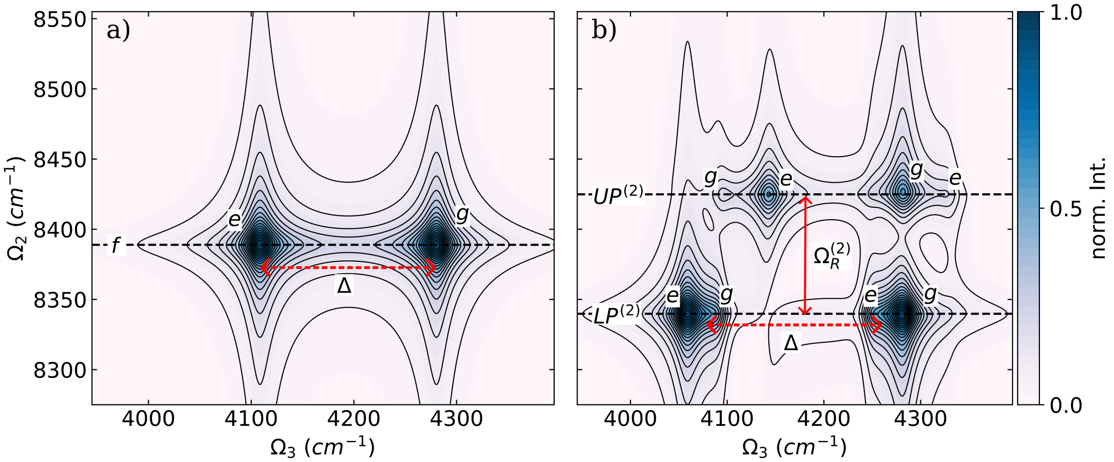

FIG. S3. Absolute value of the normalized DQC spectra of a single HF molecule a) without a cavity and b) coupled to a single cavity mode with $\omega_c = 4108\,\mathrm{cm}^{-1}$. The coupling strength λ_c is 0.03 au and the dephasing γ is $10\,\mathrm{cm}^{-1}$. The black horizontal dashed lines mark the energy of the final states, and all signals are labeled e and g, indicating that the initial state is the ground state or an intermediate state. The red lines with arrows highlight relevant energy differences. The signals were obtained using the full CBO-HF ansatz.

of only three resonances. In addition to the two resonances corresponding to the final states $UP^{(2)}$ and $LP^{(2)}$, there is a third resonance on the Ω_2 axis associated with the final state f_2. Since this state is formed by the simultaneous single excitation of stretching modes ($|11\rangle$), it cannot exist in the case of a single molecule and is only visible when the two molecules interact with the cavity mode. Without the cavity interaction, f_2 is not visible in the DQC spectra due to a cancellation of the Liouville diagrams, since the corresponding energy splitting is harmonic in nature. In the cavity, however, the harmonic energy splitting is broken by the light-matter interaction, which introduces additional anharmonicity into the system. The same situation is observed for the formation of the state $MP^{(2)}$ in the case of $\omega_c = \omega_1$ discussed in the main manuscript.

In Fig. S5 the comparison between the DQC signals based on the full CBO-HF surface, the linear CBO-HF surface, and the extended Tavis-Cummings (ETC) surfaces for the two-molecule case is shown as difference spectra $\Delta\mathcal{S}$. In both difference spectra the peaks corresponding to the final state $LP^{(2)}$ are the most affected. These peaks are red-shifted by approximately $20\,\mathrm{cm}^{-1}$ to $30\,\mathrm{cm}^{-1}$ in Ω_2 and approximately $10\,\mathrm{cm}^{-1}$ to $20\,\mathrm{cm}^{-1}$ in Ω_3 when

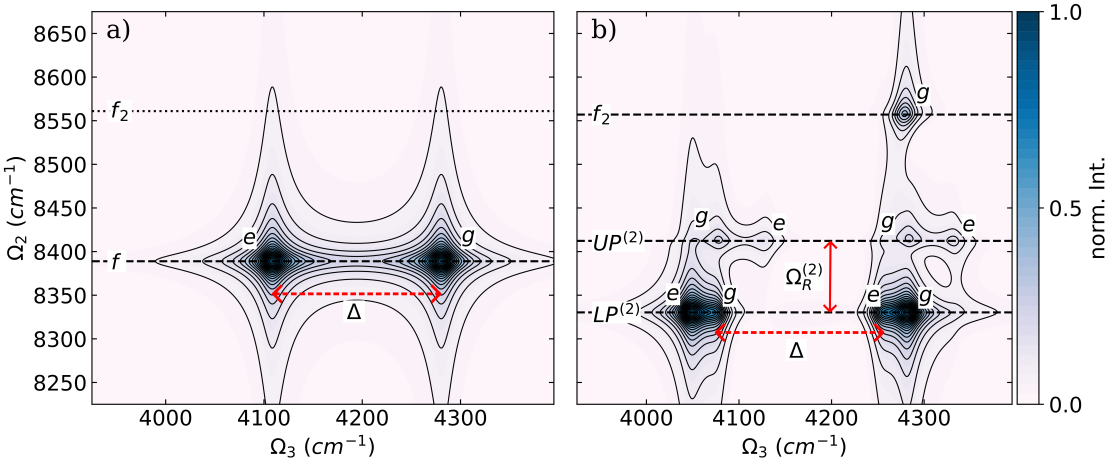

FIG. S4. Absolute value of the normalized DQC spectra of two parallel oriented HF molecules a) without a cavity and b) coupled to a single cavity mode with $\omega_c = 4108\,\mathrm{cm}^{-1}$. The coupling strength λ_0 is 0.03 au and the dephasing γ is $10\,\mathrm{cm}^{-1}$. The black horizontal dashed lines mark the energy of the final states, and all signals are labeled e and g, indicating that the initial state is the ground state or an intermediate state. The red lines with arrows highlight relevant energy differences. The signals were obtained using the full CBO-HF ansatz.

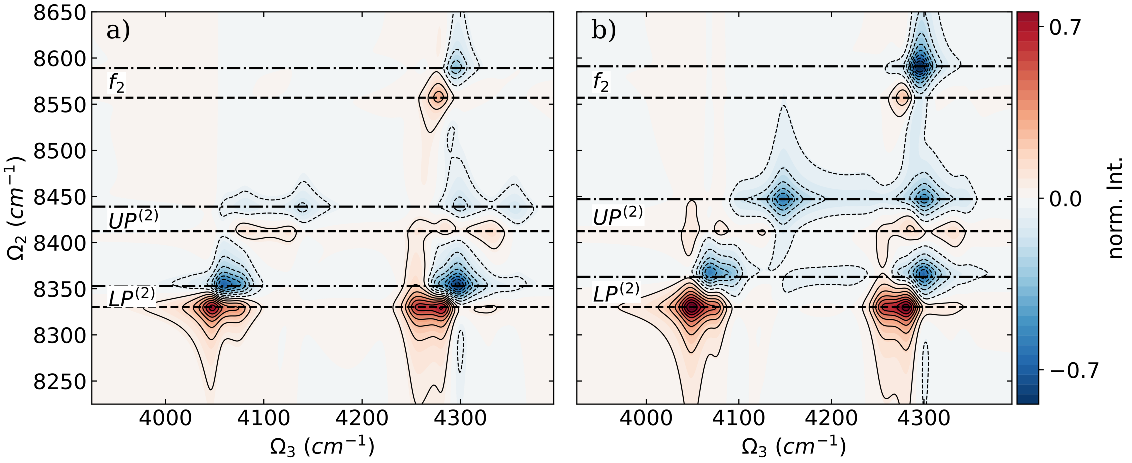

FIG. S5. Difference $\Delta\mathcal{S}$ of the DQC signal of two parallel HF molecule coupled to a photon mode with $\omega_c = 4108\,\mathrm{cm}^{-1}$ between a) full CBO-HF and linear CBO-HF and b) full CBO-HF and ETC. Individual DQC spectra are normalized, and the absolute value is used to calculate the difference. The coupling strength λ_c is 0.03 au for both frequencies and the dephasing γ is $10\,\mathrm{cm}^{-1}$. The energies of the final states are marked with black dashed lines for the full CBO-HF case and with dashed dotted lines for the linear CBO-HF and ETC cases, respectively.

the dipole self-energy (DSE) contribution is included, see Fig. S5 a), or the self-consistent field (SCF) procedure is performed, see Fig. S5 b)). A similar but weaker red shift is observed for the peaks associated with the final state $UP^{(2)}$. The DQC signal corresponding to the final state f_2 is also red-shifted when the DSE contribution is included, while the intensity is more or less unchanged. However, without the SCF treatment, the corresponding signal is not only shifted in both Ω_2 and Ω_3, but also the intensity is much higher. This result clearly indicates the sensitivity of this f_2 state to the way the cavity is described.

S3. REAL AND IMAGINARY PARTS OF THE DQC SPECTRA

The real (Re) and imaginary (Im) parts of all discussed DQC spectra are shown in Fig. S6, Fig. S7 ,Fig. S8, and Fig. S9 using the full CBO-HF energy expectation values and a coupling strength λ_c of 0.03 au.

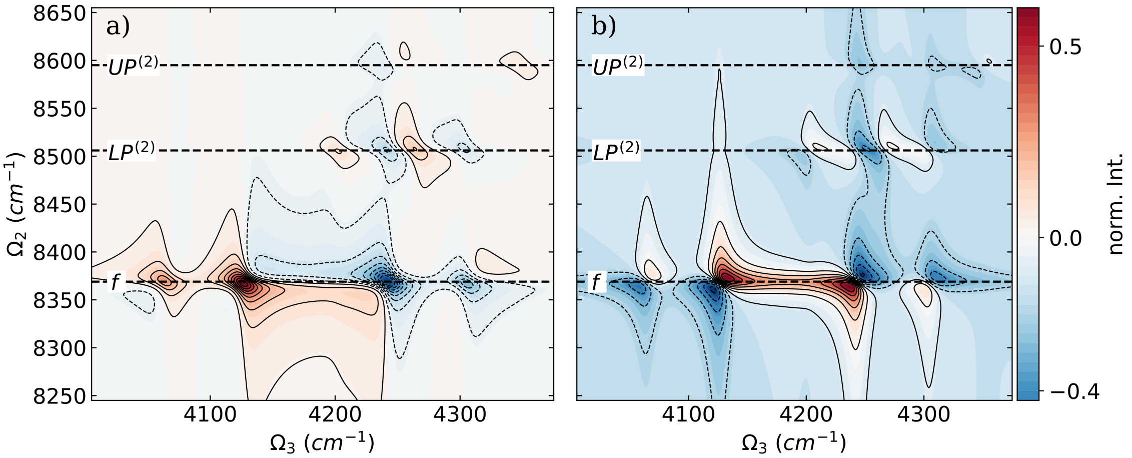

FIG. S6. a) Real (Re) part and b) imaginary (Im) part of the DQC signal of a single HF molecule coupled to a photon mode with $\omega_c = 4281\,\mathrm{cm}^{-1}$. Both are normalized with respect to the absolute value of the DQC signal. The coupling strength λ_c is 0.03 au and the dephasing γ is $10\,\mathrm{cm}^{-1}$. The black horizontal dashed lines mark the energy of the final states. The signals were obtained using the full CBO-HF ansatz.

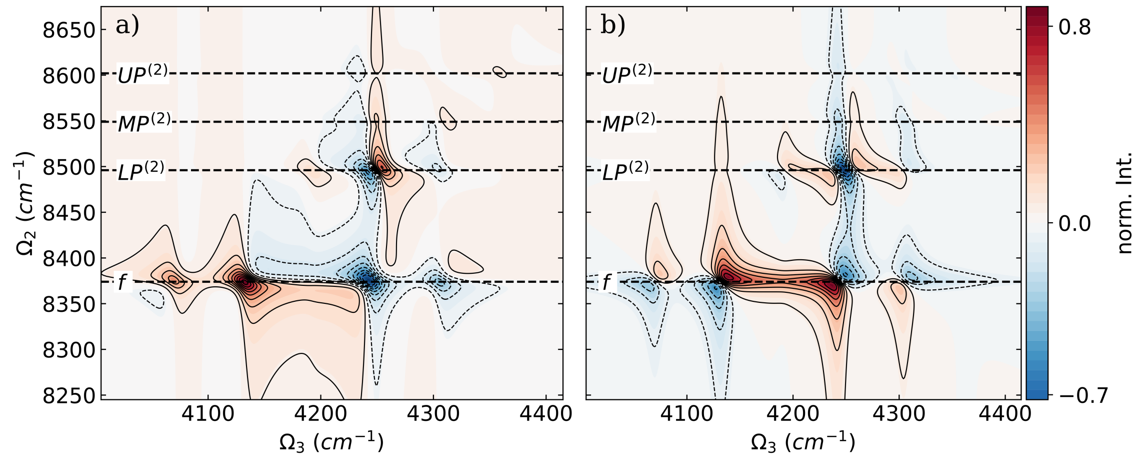

FIG. S7. a) Real (Re) part and b) imaginary (Im) part of the DQC signal of two parallel HF molecules coupled to a photon mode with $\omega_c = 4281\,\mathrm{cm}^{-1}$. Both are normalized with respect to the absolute value of the DQC signal. The coupling strength λ_c is 0.03 au and the dephasing γ is $10\,\mathrm{cm}^{-1}$. The black horizontal dashed lines mark the energy of the final states. The signals were obtained using the full CBO-HF ansatz.

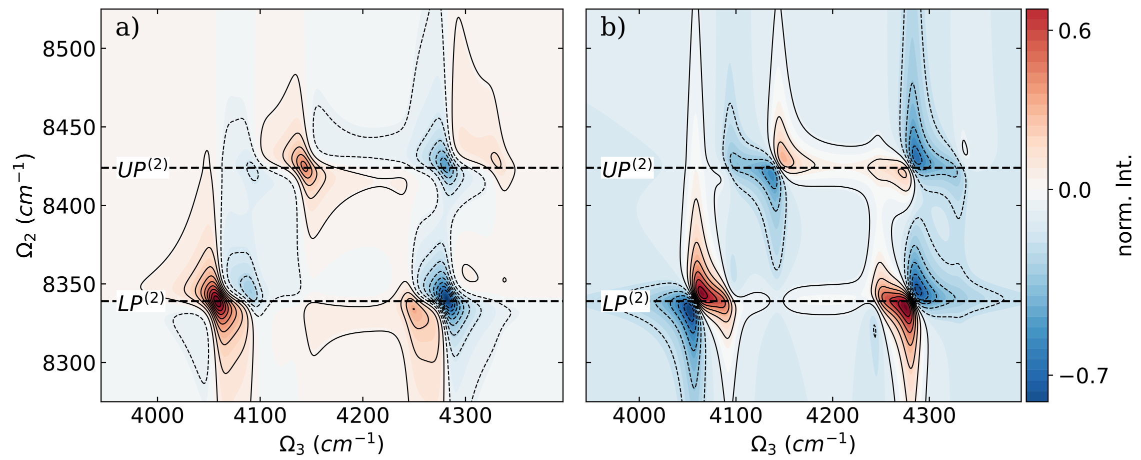

FIG. S8. a) Real (Re) part and b) imaginary (Im) part of the DQC signal of a single HF molecule coupled to a photon mode with $\omega_c = 4108\,\mathrm{cm}^{-1}$. Both are normalized with respect to the absolute value of the DQC signal. The coupling strength λ_c is 0.03 au and the dephasing γ is $10\,\mathrm{cm}^{-1}$. The black horizontal dashed lines mark the energy of the final states. The signals were obtained using the full CBO-HF ansatz.

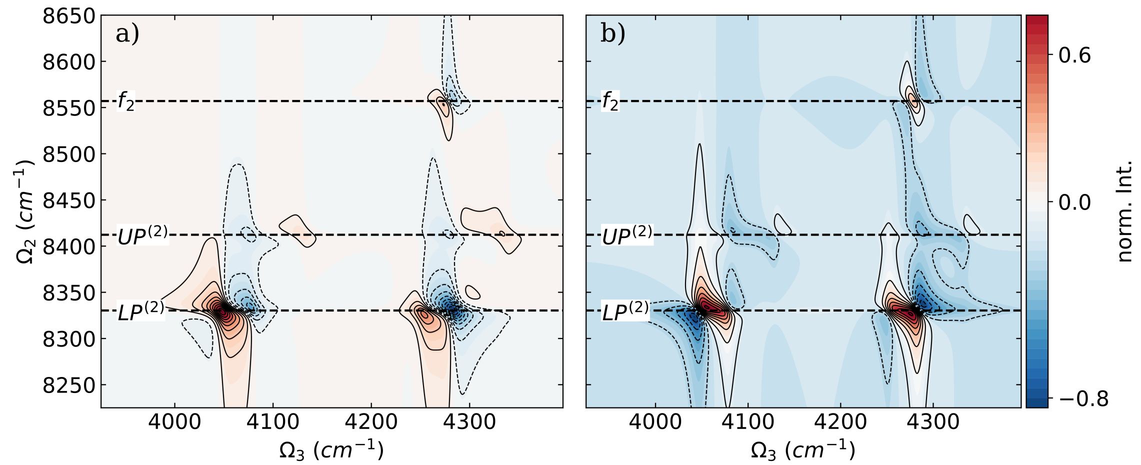

FIG. S9. a) Real (Re) part and b) imaginary (Im) part of the DQC signal of two parallel HF molecules coupled to a photon mode with $\omega_c = 4108\,\mathrm{cm}^{-1}$. Both are normalized with respect to the absolute value of the DQC signal. The coupling strength λ_c is 0.03 au and the dephasing γ is $10\,\mathrm{cm}^{-1}$. The black horizontal dashed lines mark the energy of the final states. The signals were obtained using the full CBO-HF ansatz.
